# Multidetector CT Improving Surgical Outcomes in Breast Cancer (MISO BC)

**DOI:** 10.1186/bcr3696

**Published:** 2014-11-03

**Authors:** J Cox, H Hancock, J Spratt, H Close, CM Lee, U Mohammed, J Mason

**Affiliations:** 1 County Durham and Darlington NHS Foundation Trust, Durham, UK; 2 Durham University, Durham, UK

## Introduction

This multicentre randomised controlled trial investigated whether a computed tomography (CT) scan of the axilla could more accurately assess whether the axillary lymph nodes were involved with malignancy in patients with newly diagnosed breast cancer and therefore influence surgical decision-making with regard to axillary surgery.

## Methods

Patients with newly diagnosed breast cancer (via screening and symptomatic routes) at two NHS Trusts in the North East of England were recruited and randomised in equal numbers. Both groups received routine diagnostic and surgical care (usual care). In addition, one group received a CT scan of their axilla on the same side as the breast cancer.

## Results

The study recruited 297 patients, of whom 291 contributed to findings. CT scan-guided care did not result in a change in the need for a second operation, with about 20% of both groups needing further surgery. Patients within the two groups were similar before treatment, had similar types and grade of cancer, experienced similar pattern complications and reported similar experiences of care.

## Conclusion

New diagnostic imaging technologies regularly enter NHS centres of excellence as research tools. It is important these are evaluated rigorously before becoming routine care. In patients newly diagnosed with breast cancer, CT-augmented diagnosis of cancer in the axilla was not found to improve surgical outcomes or patient experience.

